# Molecular detection of *Coxiella burnetii* in small ruminants and genotyping of specimens collected from goats in Poland

**DOI:** 10.1186/s12917-021-03051-0

**Published:** 2021-10-28

**Authors:** Agnieszka Jodełko, Monika Szymańska-Czerwińska, Jolanta Grażyna Rola, Krzysztof Niemczuk

**Affiliations:** 1 Department of Cattle and Sheep Diseases, National Veterinary Research Institute, Pulawy, Poland; 2 Department of Hygiene of Food of Animal Origin, National Veterinary Research Institute, Pulawy, Poland

**Keywords:** Q fever, *Coxiella burnetii*, Genotyping, Small ruminants, Poland, MLVA, MST

## Abstract

**Background:**

*Coxiella burnetii* is the etiological agent of Q fever, a zoonosis affecting many animal species including sheep and goats. The aims of this study were to evaluate the shedding of *Coxiella burnetii* in small ruminant herds and to identify the pathogen’s genotypes and sequence types (STs) using multiple-locus variable number tandem repeat analysis (MLVA) and multispacer sequence typing (MST) methods.

**Results:**

Overall, 165 samples from 43 herds of goats and 9 flocks of sheep were collected including bulk tank milk (BTM), individual milk samples, vaginal swabs, tissue sections from stillborn kids, feces and placentas. These were tested by real-time PCR targeting the IS*1111* element. *C. burnetii* infection was confirmed in 51.16% of the herds of goats and 22.2% of the flocks of sheep. Six out of nine samples originating from goats were successfully genotyped using the MLVA method. The presence was confirmed of two widely distributed MLVA genotypes (I and J) and genotype PL1 previously reported only in cattle. Only one sequence type (ST61) was identified; however, the majority of specimens represented partial STs and some of them may belong to ST61. Other partial STs could possibly be ST74.

**Conclusion:**

This study confirmed the relatively common occurrence of *Coxiella burnetii* in small ruminant herds in Poland. Interestingly, all genotyped samples represent cattle-associated MLVA genotypes.

## Background

Q fever is a worldwide zoonotic disease caused by *Coxiella burnetii.* It is an intracellular, Gram-negative bacterium with a reservoir in a wide range of domesticated and wild animals [1–4]. Cattle and small ruminants (sheep and goats) are commonly known as shedders of *C. burnetii* and are described as primary sources of its transmission to humans [5, 6].

Infection in small ruminants is generally subclinical, although it can trigger stillbirth and late abortion, metritis, milk yield losses as well as delivery of weak offsprings with low body weight [7–10]. The placenta and birth fluids of infected animals contaminate the environment and are a significant threat to the agricultural industry and public health. Q fever outbreaks in humans are often related to small ruminants [11, 12], and such was the case for the largest outbreak of the disease, which took place in the Netherlands from 2007 to 2010 [13].

People become infected mainly by inhalation of bacteria-contaminated aerosols, which can be spread by wind over long distances [14], or via direct contact with infected animals. Infections via consumption of contaminated milk were also described [15, 16], although the possibility of oral transmission is still under debate and requires further investigation [17]. Considering the multi-route transmission of *C. burnetii,* very low infectious dose and the long persistence of the bacteria in the environment, maintaining surveillance of this zoonotic agent seems to be imperative.

Q fever is a notifiable disease in Poland. The National Surveillance Programme for the disease executed by the Veterinary Inspectorate and fully funded by the government has been ongoing since 2010. The programme is mandatory for small ruminants and cattle older than 12 months. Veterinary inspectors for a given county have to collect the appropriate number of blood samples to guarantee detection of seroconversion in the area with a 95% confidence level, and work from a baseline assumption of 20% infection prevalence in the county. The surveillance program requires that samples are collected from randomly selected animals. If in a herd larger than 100 animals, two abortions occur in a month or three in a year or the number of abortions exceeds 4% of the total number of animals in the herd or flock, it is recommended to collect blood specimens from all the animals being reared together. If the presence of specific antibodies is confirmed, samples for molecular analysis by real-time PCR should be sent to the National Reference Laboratory for Q fever Disease (NRL). A positive real-time PCR result obtained in the NRL definitively confirms the outbreak of Q fever in the herd.

The stock of goat and sheep in Poland has drastically declined over recent decades and it is much smaller than that of cattle. The number of sheep in the country was estimated at 4.2 million animals in the 1990s but has decreased to 268,000 in 2019. Similarly to sheep, the number of goats dropped from 141,000 in 2005 to 44,000 in 2019 [18]. However, this trend may reverse because goat and sheep products have been gaining popularity in recent years. Goat and sheep production in Poland is by smallholder farmers – 81.5% of herds of goats and 41.7% of flocks of sheep consisted of 4 animals or fewer in 2010 [19]. Milk production is the predominant use of goats, while sheep are primarily raised for meat and milk. The highest number of goats are farmed in Lesser Poland (5927), followed by Greater Poland (5494) and Subcarpathia (4598). Sheep farming is an element of folk and pastoral culture, especially in the Lesser Poland Voivodeship, where the largest population of these animals lives (76,550). Other regions with high numbers of sheep are Podlaskie (26,821) and Greater Poland (22,884) [18].

The epidemiological data regarding coxiellosis in small ruminants in Poland are scarce. A seroepidemiological study carried out in 2007 did not reveal the presence of *C. burnetii* antibodies in the tested herds of Polish goats [20]. In contrast, research on small ruminants in 2016–2017 demonstrated herd-level seroprevalence of 27.27% [21]. The presence of seropositive animals and limited nature of the data about the prevalence and genotypic diversity of *C. burnetii* in small ruminants were the reasons to undertake further research. Genotypic characterisation is useful for surveillance of the circulation of *C. burnetii* strains and establishing the potential connection between genotypes and virulence of the strains. Genotyping tools such as MLVA and MST allow the outbreaks to be traced back and the genotypes of *C. burnetii* present in local areas to be determined [22–24].

This study was performed to evaluate the shedding of *C. burnetii* in small ruminant herds in Poland based on a real-time PCR test. Genotypes of the pathogen present in the tested specimens were also identified using MLVA and MST methods.

## Results

**Fig. 1:**
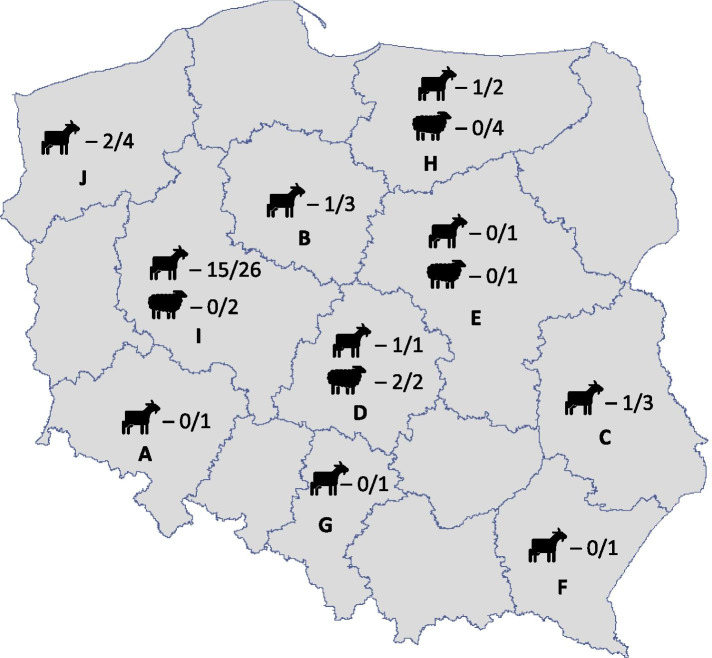
Number of positive and tested goat herds and flocks of sheep in Polish voivodeships. Legend: Voivodeships: A – Lower-Silesia, B – Kuyavia-Pomerania, C – Lublin, D – Łódź, E - Masovia, F – Subcarpathia, G - Silesia, H – Warmia-Masuria, I – Greater Poland, J – West Pomerania

The shedding of *C. burnetii* was detected in 51.16% (95% confidence interval [CI]: 35.5–66.7) of herds of goats and 22.2% (95% CI 2.8–60.0) of flocks of sheep. Shedders were detected in goat herds located in Kuyavia-Pomerania (1/3), Lublin (1/3), Łódź (1/1), Greater Poland (15/26), Warmia-Masuria (1/2) and West Pomerania (2/4) voivodeships, while positive sheep flocks were noted only in Łódź (2/2) (Fig. 1). Detailed real-time PCR result data at herd level are presented in Table 1.

**Table 1:** Results of real-time PCR at herd level

Type of tested sample	No. of tested herds of goats	No. of positive herds of goats	No. of tested flocks of sheep	No. of positive flocks of sheep
individual milk	2	0	1	1
BTM	35	19	2	0
vaginal swab	2	1	4	0
individual milk and vaginal swab	2	0	1	1
feces and vaginal swab	0	0	1	0
individual milk, vaginal swab, placenta	1	1	0	0
individual milk, vaginal swab, placenta and stillborn kids	1	1	0	0
**Total**	**43**	**22**	**9**	**2**

BTM specimens accounted for the highest number of positive samples (19/37). DNA of *C. burnetii* was detected in 3/29 individual milk samples, 2/74 vaginal swabs and 1/14 placentas. All tested feces samples were negative, in contrast to sections from internal organs (the liver, lungs, heart, and spleen) of stillborn kids (twins), which were all positive in the real-time PCR test.

Genotyping using the MST technique revealed that the majority of tested samples belonged to ST61 (Table 2). Incomplete allelic combinations established for two specimens indicated that they may have represented one of the two sequence types ST61 or ST74.

**Table 2:** Results of genotyping using the MST method

No.	Herd ID	Voivodeship	Type of sample	Ct real-time PCR	Alleles identified in spacers^b^										Sequence type
					Cox 2	Cox 5	Cox 18	Cox 20	Cox 22	Cox 37	Cox 51	Cox 56	Cox 57	Cox 61	
1.	B1	Kuyavia-Pomerania	BTM	29.27	3	–	6	1	–	10	4	10	–	5	61^a^
2.	C1	Lublin	tissue	29.28	3	2	6	1	5	10	4	10	6	5	61
3.	I1	Greater Poland	BTM	30.94	3	2	–	–	5	10	4	10	6	5	61/74^a^
4.	I2	Greater Poland	BTM	31.18	3	2	6	–	5	10	–	10	–	5	61^a^
5.	I3	Greater Poland	BTM	31.19	3	2	6	–	5	10	4	10	6	5	61^a^
6.	I4	Greater Poland	BTM	29.76	3	2	6	1	5	10	4	10	–	5	61^a^
7.	I5	Greater Poland	BTM	29.36	3	2	6	1	5	10	4	10	–	5	61^a^
8.	I6	Greater Poland	BTM	30.98	3	–	–	1	5	10	–	10	–	5	61/74^a^
9.	J1	West Pomerania	BTM	29.6	3	2	6	1	–	10	–	10	–	5	61^a^

^a^ Sequence type established based on incomplete allelic profile

^b^According to an online database [25]

Three MLVA genotypes (I, J and PL1) were identified in the tested goat samples. Genotype J with the numbers of short tandem repeats (STRs) 6–13–2-7-9-10 for loci Ms23-Ms24-Ms27-Ms28-Ms33-Ms34 occurred most commonly and was present in 4 herds from two voivodeships. Genotype I (6–13–2-7-9-9) was detected in one herd from Kuyavia-Pomerania, while PL1 (6–14–2-7-9-9) occurred in a herd located in Lublin. Genotypes J and PL1 differed from each other in the number of STRs in two loci (Ms24 and Ms34), whereas they differed from genotype I only in the number of these in one locus. For three of the tested herds only partial allelic profiles were obtained, and determination of MLVA genotypes was therefore not possible. Detailed results of genotyping using the MLVA technique are shown in Table 3.

**Table 3:** Summary of the results of genotyping by MLVA

No.	Herd ID	Voivodeship	Type of sample	Ct real-time PCR	No. of STRs in locus						MLVA genotype ^a^
					Ms 23	Ms 24	Ms 27	Ms 28	Ms 33	Ms 34	
1.	B1	Kuyavia-Pomerania	BTM	29.27	6	13	2	7	9	9	I
2.	C1	Lublin	tissue	29.28	6	14	2	7	9	9	PL1
3.	I1	Greater Poland	BTM	30.94	6	13	2	7	9	10	J
4.	I2	Greater Poland	BTM	31.18	–	27	2	7	9	–	ND
5.	I3	Greater Poland	BTM	31.19	–	13	–	–	9	10	ND
6.	I4	Greater Poland	BTM	29.76	–	13	2	7	–	10	ND
7.	I5	Greater Poland	BTM	29.36	6	13	2	7	9	10	J
8.	I6	Greater Poland	BTM	30.98	6	13	2	7	9	10	J
9.	J1	West Pomerania	BTM	29.6	6	13	2	7	9	10	J

ND – not determined

^a^ according to nomenclature proposed by Tilburg [26]

**Fig. 2:**
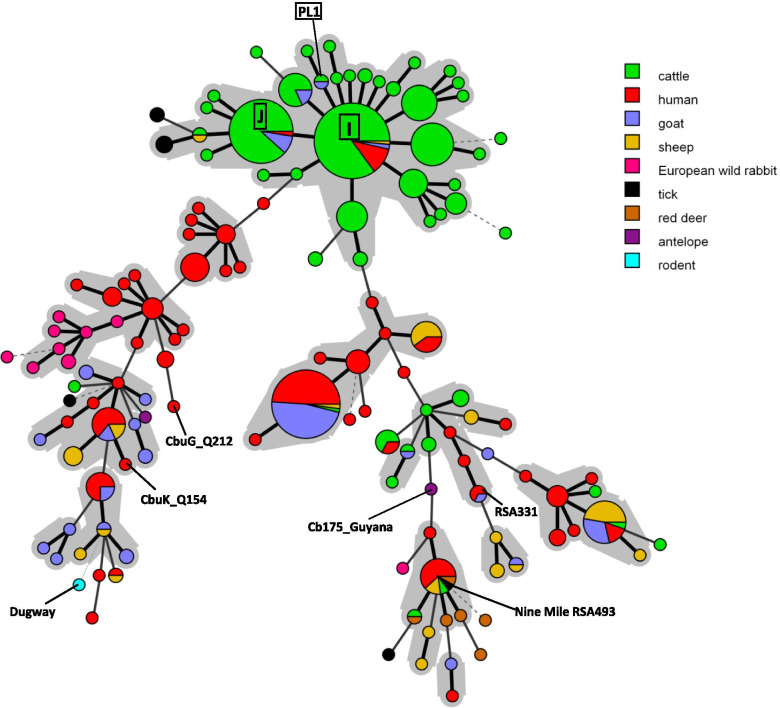
Minimum spanning tree showing the relationship between MLVA genotypes with reference to the host species. Legend: Minimum spanning tree shows genotypes identified in this study (I, J and PL1), collected in the database [27] and reported elsewhere [24, 28–30]. Six reference strains of *C. burnetii*: CbuG_Q212, CbuG_Q154, Cb175_Guyana, Dugway, Nine Mile RSA493 and Henzerling RSA331 are indicated. Genotypes connected by a gray background differ in only one marker from each other and may represent microvariants of one founder genotype

**Fig. 3:**
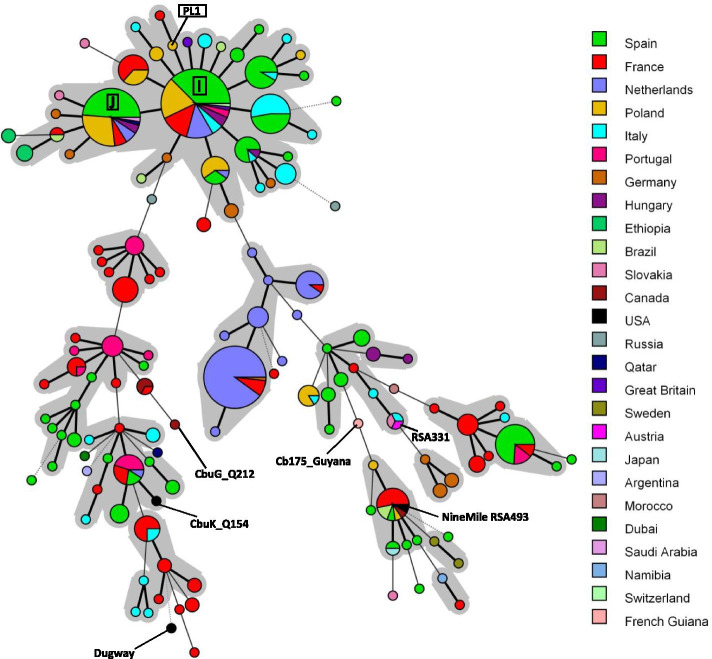
Minimum spanning tree showing the relationship between MLVA genotypes with reference to the geographical orgin. Legend: Minimum spanning tree shows genotypes identified in this study (I, J and PL1), collected in the database [27] and reported elsewhere [24, 28–30]. Six reference strains of *C. burnetii*: CbuG_Q212, CbuG_Q154, Cb175_Guyana, Dugway, Nine Mile RSA493 and Henzerling RSA331 are indicated. Genotypes connected by a gray background differ in only one marker from each other and may represent microvariants of one founder genotype

Figure 2 and Fig. 3 present minimum spanning trees showing the relationships between MLVA genotypes identified in this study, published in the database [27], and reported elsewhere [24, 28–30]. Only complete MLVA-6 genotypes were included.

Clustering of the MLVA genotypes using the minimum spanning tree method showed a high genetic similarity between all of those identified. It is noteworthy that the samples tested in this study present genotypes previously identified mainly in cattle. These predominantly cattle-hosted genotypes had been detected in many countries, including Poland.

**Fig. 4:**
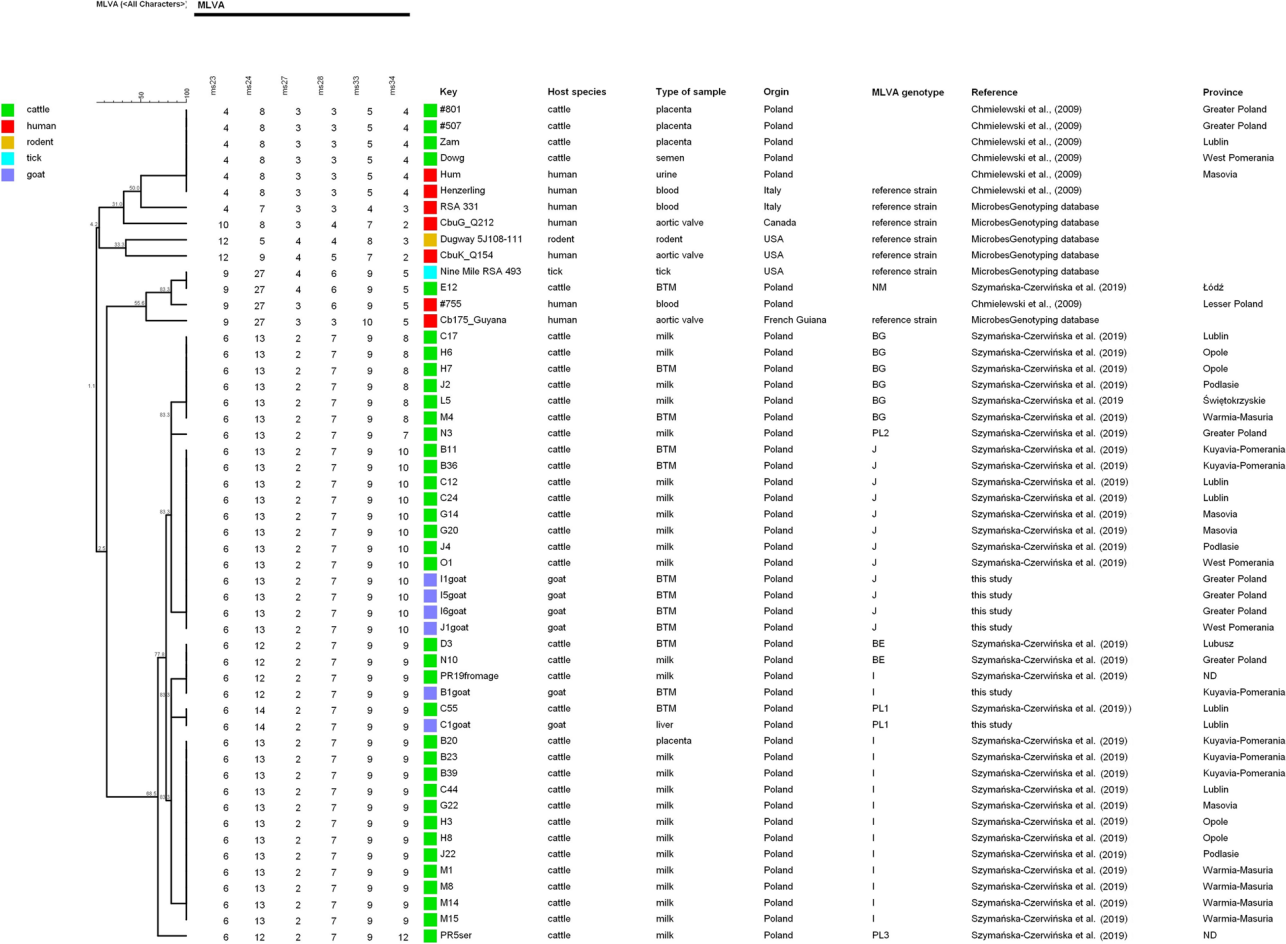
UPGMA cluster analysis of *C. burnetii* genotypes identified in Poland using the MLVA technique. Legend: ND – not determined, lack of data about localization

The unweighted pair group method with arithmetic mean (UPGMA) cluster analysis of the MLVA data revealed that the genotypes identified in this research and the genotypes of the strains analysed by Chmielewski et al. [31] are in different clusters (Fig. 4). All specimens tested in this study belong to the same cluster as genotypes identified previously in Polish cattle [32] and exhibit a high level of similarity.

## Discussion

The investigation performed in this study revealed that *C. burnetii* infections are quite common in the small ruminant population in Poland. Pre-existing data on the epizootic situation of Q fever in Polish goats and sheep are very limited, so it is difficult to compare the dynamic of the situation over the years. The first outbreak of Q fever was recorded in 1956 in a village near Gorlice (south-eastern Poland) and the source of human infection were sheep imported from Romania [33]. In 1985, an outbreak of the disease was confirmed in European bison from the Białowieża primeval forest (eastern Poland) and in cattle and sheep kept in nearby villages [34]. Data from 2007 published by Czopowicz et al. [20] did not reveal the presence of *C. burnetii* antibodies in tested herds of Polish goats. Nevertheless, the most recent research carried out on small ruminants in 2016–2017 demonstrated herd-level seroprevalence of 27.27% [21]. It is comparable with results published by Wolf et al. [35] who confirmed the presence of specific antibodies in 28.2% of German herds and detected *C. burnetii* DNA in the genital swabs of 14.1% by real-time PCR. Similar seroprevalence levels were reported in herds of goats (28.8%) and flocks of sheep (37.5%) by Portuguese researchers [36].

The real-time PCR test confirmed the presence of *C. burnetii* DNA in 51.16% of tested herds of goats and 22.2% of flocks of sheep. A similar percentage of positive sheep flocks (22%) was detected in northern Spain by BTM analysis [37], while in Portugal it was significantly lower (5.1%) [38]. In Belgium, the prevalence in herds of goats ranged between 6.3 and 12.1% (data for December 2009–March 2013) [39]. A considerable difference was identified between the seroprevalence levels reported in previous studies and the high percentage of positive herds found in this research. Some studies showed that a significant percentage of seronegative animals shed the bacteria without any clinical symptoms of the disease, and this factor may have contributed to the divergence [40, 41]. Furthermore, the findings about shedding have to be seen in light of some study limitations. Only a small number of invited farmers agreed to take part in the study. Due to low response rate, specimens were collected from only 10 out of 16 Polish provinces and the number of examined herds and flocks was limited. Moreover, the majority of tested and positive herds of goat (15/26) were located in Greater Poland voivodeship, therefore the high percentage of positive herds may not reflect the epizootic situation in the whole country. A high number of infected herds does not result in a high number of Q fever cases in humans. According to reports published by The National Institute of Public Health - National Institute of Hygiene, the last cases (*n* = 4) of the disease were confirmed in Poland in 2019 [42]. It cannot be excluded that the number of cases is underreported, due, among other factors, to the nonspecific, flu-like symptoms of the acute form of the disease.

MLVA and MST are methods commonly used for *Coxiella burnetii* genotyping due to their high discriminatory power and possibility to be applied directly to DNA samples without previous cultivation of the bacteria. In this study, only partial MLVA profiles were obtained for three of the tested herds. This could have been caused by low quality and load of bacterial DNA. Lack of amplicons for the Ms23 and Ms33 markers may also suggest the presence of mutations, including insertions of the IS1111 element, in these regions. The problem was reported in other studies and it is observed especially in evaluation of small ruminant samples [24, 43, 44]. Genotyping using the MLVA technique revealed that the tested specimens belong to three genotypes: PL1, I and J. The latter two are widely distributed worldwide, but usually identified in cattle-derived samples. Genotypes I and J were found in specimens collected from asymptomatic cows in Hungary [45] as well as in Spanish cattle herds with abortions and fertility disorders [46] and also in cattle in Poland [32]. They were the predominant genotypes of *C. burnetii* in 116 tested consumer milk products available on the market in 28 countries [43]. Genotype I was also found in samples collected from goats and sheep in the Netherlands and France [27]. An interesting observation is that genotype PL1 had been identified for the first time in a BTM sample from a herd of cattle located in the same province of Poland as the one in which a goat tested positive for it [32], which indicates local spreading of the genotype in this area.

Many research publications report genotype clustering by host species, which indicates host specificity of *C. burnetii* in domestic ruminants and wildlife [29, 47]. Surprisingly, all samples collected from small ruminants in this study represent cattle-associated MLVA genotypes. These genotypes were identified as the most prevalent in recent research on the genetic diversity of *C. burnetii* in Polish dairy cattle [32]. It can be assumed that the pathogen was transmitted from cattle herds to small ruminants; such cross infection within the ruminant population has been reported sporadically [48]. In Poland, the population of small ruminants is several times smaller than cattle (310,000 and 6,278,000 animals, respectively) [18]. Sheep and goat farms may be surrounded by cattle farms; moreover, these species are often kept together. In the spring and summer seasons small ruminants graze on pastures, where they may come into contact with animals from other herds. Grazing also increases the risk of infestation by ticks harbouring *C. burnetii* because the pathogen is present in the population of these arthropods in Poland [49, 50], although no distinction was made between *C. burnetii* and *Coxiella*-like endosymbionts in the studies which established this presence. Animals at pasture are also at risk of infection by airborne transmission, especially in the dry, windy and hot weather which Poland frequently experiences in summer. Based on the result of a mathematical modeling study, it may be assumed that airborne transmission plays the major role in the regional spread of *C. burnetii* [51]. As goats and sheep are imported to Poland on a very small scale, the significance of the possible introduction of novel *C. burnetii* genotypes specific to small ruminants with imported animals seems to be marginal. In contrast, the international and local cattle trade is high volume. The factors outlined here might facilitate the spreading of cattle-derived *C. burnetii* strains to and among herds of goats.

Molecular characterisation using the MST method revealed that tested samples belong to ST61, regardless of their MLVA genotypes. This sequence type shows the highest similarity to ST20. To date, ST61 has been described in Polish dairy cattle and also in goats and cattle from Belgium [32, 44]. Mioni et al. [28] performed a phylogenic analysis with the six intergenic spacers which showed a close relationship between the novel sequence type of Brazilian strains (ST74) and ST20 and ST61.

Analyses revealed that the genotypes of *C. burnetii* strains circulating in Polish small ruminants are different from those of strains isolated from Q fever outbreaks in the twentieth century [31]. Then, an ovine-derived strain of ST16 was isolated from the first outbreak of Q fever in Poland from a patient with an acute flu-like form of the disease; however, the current study did not confirm the presence of ST16 strains in tested herds. This sequence type was recently identified in a BTM specimen collected from a herd of cattle in Łódź voivodeship, but the presence of ST16 has not been detected in other regions [32]. In this research, positive samples originating from this region were not genotyped due to high Ct values. Moreover, the specimens tested in this study belong to different a sequence type to ST3 and ST6, which were identified by analysis of 6 out of 10 intergenic spacers on two dairy cattle farms in central and eastern Poland [52]. It should be noted that few samples were genotyped, therefore the results may not be representative for the general small ruminant population. The presence of genotypes associated with small ruminants is highly probable, although they were not identified in this study.

## Conclusions

This research provides data about the shedding and genotypic diversity of *C. burnetii* in herds of small ruminants in Poland. The presence of *C. burnetii* infection was confirmed in a high number of tested herds, however a large-scale survey should be carried out to assess the prevalence in the population. Examination of nine *C. burnetii* samples originating from goats revealed the presence of three MLVA genotypes (I, J and PL1) and one sequence type (ST61). The MLVA and MST profiles identified in this research were different to profiles of the strains involved in the Q fever outbreak in the Netherlands. Considering the low number of specimens genotyped and their single-species origin (goats), further studies should be performed to fully investigate the genotypic diversity of *C. burnetii* strains circulating in Polish small ruminants.

The results of this research confirm the need to continue the public information campaign about Q fever, especially among occupationally exposed people. Moreover, active surveillance, strain genotyping and integration of this type of data at national and global level should be performed to facilitate rapid tracing of the origins of Q fever outbreaks.

## Methods

A total of 165 samples from 43 herds of goats and 9 flocks of sheep were collected between January 2016 and September 2017 in 10 out of 16 Polish voivodeships. Specimens were taken from randomly selected herds in Lower Silesia (A), Kuyavia-Pomerania (B), Lublin (C), Łódź (D), Masovia (E), Subcarpathia (F), Silesia (G), Warmia-Masuria (H), Greater Poland (I) and West Pomerania (J). Animal health status was unknown, but none of the farmers reported problems with abortions or stillbirths in the tested individuals. Various types of specimen were subjected to study, depending on availability. Bulk tank milk samples were collected from 2 flocks and 35 herds. Additionally 29 individual milk specimens, 74 vaginal swabs, 8 tissue sections from 2 stillborn kids, 3 feces and 14 placenta samples collected from 62 goats from 8 herds and 21 sheep from 7 flocks were tested. Figure 1 presents detailed data about the number of tested and positive herds and flocks in each voivodeship. Vaginal swabs were taken from animals within 8 days of parturition. All collected specimens were stored and transported to the laboratory at 5 ± 3 °C and subjected to analysis within 48 h.

DNA extraction was performed using a QIAamp DNA Mini Kit (Qiagen, Germany) according to the instructions of the manufacturer of the real-time PCR to be used next. DNA aliquots were stored at − 20 °C until use. The qualitative real-time PCR, detecting the IS*1111* transposon-like repetitive sequence of the *C. burnetii* genome, was performed in a 7500 Fast Real-Time PCR system v2.3 (Applied Biosystems, USA) using an ADIAVET™ COXIELLA REAL TIME kit (BioX Diagnostics, France). A panel of required positive and negative controls was included in each run. Only samples presenting a typical amplification curve with a cycle threshold (Ct) value below 36.0 were considered positive. The real-time PCR procedure was validated under laboratory conditions and accredited by the Polish Centre for Accreditation.

According to Polish regulations, an animal is considered positive if shedding of *C. burnetii* is confirmed by real-time PCR in at least one specimen collected from the individual. A Q fever outbreak is confirmed in the herd if the presence of *C. burnetii* DNA is detected by real-time PCR in at least one animal from it.

In the next step, DNA specimens obtained from goats were used for genotyping by MLVA and MST. In total, 9 DNA samples (8 BTM and 1 tissue) with the highest purity of DNA and Ct values below 32 were selected for genotyping. Unfortunately, the quality and DNA load in positive samples collected from sheep were too low for them to be included in further analyses.

MST was performed as described by Glazunova et al. [22] for 10 spacers: Cox2, Cox5, Cox18, Cox20, Cox22, Cox37, Cox51, Cox56, Cox57 and Cox61. Raw sequence data were assembled using Geneious Pro 8.0 software (Biomatters, New Zealand) and sequence types were determined based on the reference MST database [25].

MLVA was performed for the panel of 6 out of 17 loci published by Arricau-Bouvery et al. [23]. Three heptanucleotide repeat markers (Ms23, Ms24 and Ms33) and three hexanucleotide repeat markers (Ms27, Ms28 and Ms34) were identified using primers published by Tilburg et al. [53] and Klaassen et al. [54]. The DNA amplification was carried out as described by Chmielewski et al. [31] in a T-Personal 48 Thermocycler (Biometra, Germany). Analysis of the amplification products was performed on an ABI 3500 Genetic Analyser and electropherograms were evaluated with GeneMapper software v4.1 (Applied Biosystems, USA). The number of repeats in each locus was extrapolated by comparing the sizes of the obtained fragments with those obtained for the reference strain Nine Mile RSA 493 in the same run. Based on in silico analysis, the established genotype of this strain contained 9–27–4-6-9-5 repeats for marker loci Ms23-Ms24-Ms-27-Ms28-Ms33-Ms34, respectively. Clustering of the obtained MLVA profiles was performed with Bionumerics v.7.6 software (Applied Maths, USA). Minimum spanning trees were constructed to show the relationships between the MLVA genotypes obtained in this study, genotypes from a database [27] and those from research publications using the same genotyping scheme [24, 28–30]. Additionally, the UPGMA method was used to create a dendrogram illustrating the genetic relationships between the MLVA profiles identified in this study and those described previously in Poland.

## Data Availability

The data used and/or analyzed during the current study are available from the corresponding author on reasonable request.

## Abbreviations

BTM: bulk tank milk
MLVA: multiple-locus variable number tandem repeat analysis
MST: multispacer sequence typing
ST: sequence type
Ct: cycle threshold
STR: short tandem repeat
UPGMA: unweighted pair group method with arithmetic mean
CI: confidence interval

## References

[1] Pinsky RL, Fishbein DB, Greene CR, Geinshemer KF (1991). An outbreak of cat-associated Q fever in the United States. J Infect Dis.

[2] Stein A, Raoult D (1999). Pigeon pneumonia in Provence. A bird-borne Q fever outbreak. Clin Infect Dis.

[3] González-Barrio D, Maio E, Vieira-Pinto M, Ruiz-Fons F (2015). European rabbits as reservoir for Coxiella burnetii. Emerg Infect Dis.

[4] Fernandez-Aguilar X, Cabezon O, Colom-Cadena A, Lavín S, López-Olvera JR (2016). Serological survey of *Coxiella burnetii* at the wildlife-livestock interface in the eastern Pyrenees, Spain. Acta Vet Scand.

[5] Angelakis E, Raoult D (2009). Q fever. Vet Microbiol.

[6] EFSA (2010). Panel on animal health and welfare (AHAW); scientific opinion on Q fever. EFSA J.

[7] Arricau Bouvery N, Souriau A, Lechopier P, Rodolakis A (2003). Experimental *Coxiella burnetii* infection in pregnant goats: excretion routes. Vet Res.

[8] van den Brom R, Van EE, Roest HI, van der Hoek W, Vellema P. *Coxiella burnetii* infections in sheep or goats: an opinionated review. Vet Microbiol 2015;181(1–2):119–129. 10.1016/j.vetmic.2015.07.011 26315774

[9] Berri M, Rousset E, Hechard C, Champion JL, Dufour P, Russo P (2005). Progression of Q fever and *Coxiella burnetii* shedding in milk after an outbreak of enzootic abortion in a goat herd. Vet Rec.

[10] Canevari J, Firestone S, Vincent G, Campbell GA, Tan T, Muleme M (2018). The prevalence of *Coxiella burnetii* shedding in dairy goats at the time of parturition in an endemically infected enterprise and associated milk yield losses. BMC Vet Res.

[11] Gilsdorf A, Kroh C, Grimm S, Jensen E, Wagner-Wiening C, Alpers K (2008). Large Q fever outbreak due to sheep farming near residential areas, Germany, 2005. Epidemiol Infect.

[12] Bellini C, Magouras I, Chapuis-Taillard C, Clerc O, Masserey E, Peduto G (2014). Q fever outbreak in the terraced vineyards of Lavaux, Switzerland. New Microbes New Infect.

[13] Roest HIJ, Ruuls RC, Tilburg JJHC, Nabuurs-Franssen MH, Klaassen CHW, Vellema P (2011). Molecular epidemiology of Coxiella burnetii from ruminants in Q fever outbreak, the Netherlands. Emerg Infect Dis.

[14] Clark NJ, Soares Magalhães RJ (2018). Airborne geographical dispersal of Q fever from livestock holdings to human communities: a systematic review and critical appraisal of evidence. BMC Infect Dis.

[15] Brown GL, Colwell DC, Hooper WL (1968). An outbreak of Q fever in Staffordshire. J Hyg (Lond).

[16] Signs K, Stobierski M, Gandhi T (2012). Q fever cluster among raw milk drinkers in Michigan, 2011. Clin Infect Dis.

[17] Koehler LM, Kloppert B, Hamann HP, El-Sayed A, Zschöck M (2019). Comprehensive literature review of the sources of infection and transmission routes of *Coxiella burnetii*, with particular regard to the criteria of "evidence-based medicine". Comp Immunol Microbiol Infect Dis.

[18] Statistics Poland. Reports about the farm animal population by species. https://bdl.stat.gov.pl/BDL/metadane/podgrupy/181?back=True# . Accessed 10 June 2021.

[19] Statistics Poland. The 2010 Agricultural Census and the Survey on Agricultural Production Methods. https://stat.gov.pl/download/cps/rde/xbcr/gus/rl_psr2010_zwierz_gosp_i_wybrane_elemy_metod_prod.pdf. Accessed 10 June 2021.

[20] Czopowicz M, Kaba J, Szaluś-Jordanow O, Nowicki M, Witkowski L, Nowicka D (2010). Prevalence of antibodies against *Chlamydophila abortus* and *Coxiella burnetii* in goat herds in Poland. Pol J Vet Sci.

[21] Szymańska-Czerwińska M, Jodełko A, Pluta M, Kowalik S, Niemczuk K (2017). Seroprevalence of *Coxiella burnetii* among domestic ruminants and horses in Poland. Acta Virol.

[22] Glazunova O, Roux V, Freylikman O, Sekeyova Z, Fournous G, Tyczka J (2005). *Coxiella burnetii* genotyping. Emerg Infect Dis.

[23] Arricau-Bouvery N, Hauck Y, Bejaoui A, Frangoulidis D, Bodier C, Souriau A (2006). Molecular characterization of *Coxiella burnetii* isolates by infrequent restriction site-PCR and MLVA typing. BMC Microbiol.

[24] Ceglie L, Guerrini E, Rampazzo E, Barberio A, Tilburg J, Hagen F (2015). Molecular characterization by MLVA of *Coxiella burnetii* strains infecting dairy cows and goats of North-Eastern Italy. Microbes Infect.

[25] Multi Spacers Typing - *Coxiella Burnetii* database. https://ifr48.timone.univ-mrs.fr/mst/coxiella_burnetii/. Accessed 20 Jan 2021.

[26] Tilburg JJ. Molecular investigation of the Q fever epidemic in the Netherlands. The largest outbreak caused by *Coxiella burnetii* ever reported [dissertation on the internet]. Nijmegen: The Radboud University. 2013. [cited 2021 Jan 21]. Available from: https://repository.ubn.ru.nl/bitstream/handle/2066/112910/112910.pdf?sequence=1.

[27] MicrobesGenotyping – MLVA Database. https://microbesgenotyping.i2bc.paris-saclay.fr/databases/view/16. Accessed 20 Jan 2021.

[28] Mioni MSR, Sidi-Boumedine K, Morales Dalanezi F, Fernandes Joaquim S, Denadai R, Reis Teixeira WS (2019). New genotypes of *Coxiella burnetii* circulating in Brazil and Argentina. Pathogens..

[29] González-Barrio D, Hagen F, Tilburg JJ, Ruiz-Fons F (2016). *Coxiella burnetii* genotypes in Iberian wildlife. Microb Ecol.

[30] Piñero A, Barandika JF, García-Pérez AL, Hurtado A (2015). Genetic diversity and variation over time of *Coxiella burnetii* genotypes in dairy cattle and the farm environment. Infect Genet Evol.

[31] Chmielewski T, Sidi-Boumedine K, Duquesne V, Podsiadły E, Thiéry R, Tylewska-Wierzbanowska S (2009). Molecular epidemiology of Q fever in Poland. Pol J Microbiol.

[32] Szymańska-Czerwińska M, Jodełko A, Zaręba-Marchewka K, Niemczuk K (2019). Shedding and genetic diversity of *Coxiella burnetii* in polish dairy cattle. PLoS One.

[33] Lutyński R (1993). First focus of Q-fever on the territory of Poland. Przegl Lek.

[34] Anusz Z, Walkowiak E, Krupa J, Kruszewska D, Rumin W, Ciecierski H. Occurence of *Coxiella burnetii* antibodies in bisons (*Bison bonasus*) from Bialowieski Primeval Forest and in cattle and sheep from villages around it. Proceedings of the 37 Annual Meeting of the European Association for Animal Production; 1991 Sep 1–6; Budapest, Hungary.

[35] Wolf A, Prüfer TL, Schoneberg C, Campe A, Runge M, Ganter M (2020). Prevalence of *Coxiella burnetii* in German sheep flocks and evaluation of a novel approach to detect an infection via preputial swabs at herd-level. Epidemiol Infect.

[36] Anastácio S, Tavares N, Carolino N, Sidi-Boumedine K, Da Silva GJ (2013). Serological evidence of exposure to *Coxiella burnetii* in sheep and goats in Central Portugal. Vet Microbiol.

[37] García-Pérez AL, Astobiza I, Barandika JF, Atxaerandio R, Hurtado A, Juste RA (2009). Short communication: investigation of *Coxiella burnetii* occurrence in dairy sheep flocks by bulk-tank milk analysis and antibody level determination. J Dairy Sci.

[38] Anastácio S, Carolino N, Sidi-Boumedine K, da Silva GJ (2016). Q fever dairy herd status determination based on serological and molecular analysis of bulk tank Milk. Transbound Emerg Dis.

[39] Boarbi S, Mori M, Rousset E, Sidi-Boumedine K, Van Esbroeck M, Fretin D (2014). Prevalence and molecular typing of *Coxiella burnetii* in bulk tank milk in Belgian dairy goats, 2009–2013. Vet Microbiol.

[40] Rousset E, Berri M, Durand B, Dufour P, Prigent M, Delcroix T (2009). *Coxiella burnetii* shedding routes and antibody response after outbreaks of Q fever-induced abortion in dairy goat herds. Appl Environ Microbiol.

[41] Rodolakis A, Berri M, Héchard C, Caudron C, Souriau A, Bodier CC (2007). Comparison of *Coxiella burnetii* shedding in milk of dairy bovine, caprine, and ovine herds. J Dairy Sci.

[42] The National Institute of Public Health - National Institute of Hygiene. Number of cases of selected infectious diseases confimed in Poland in 2019 and 2020. http://wwwold.pzh.gov.pl/oldpage/epimeld/2020/INF_20_12B.pdf . Accessed 11 June 2021.

[43] Tilburg JJ, Roest HJ, Nabuurs-Franssen MH, Horrevorts AM, Klaassen CH (2012). Genotyping reveals the presence of a predominant genotype of *Coxiella burnetii* in consumer milk products. J Clin Microbiol.

[44] Tomaiuolo S, Boarbi S, Fancello T, Michel P, Desqueper D, Grégoire F (2021). Phylogeography of human and animal *Coxiella burnetii* strains: genetic fingerprinting of Q fever in Belgium. Front Cell Infect Microbiol.

[45] Sulyok KM, Kreizinger Z, Hornstra HM, Pearson T, Szigeti A, Dán Á (2014). Genotyping of *Coxiella burnetii* from domestic ruminants and human in Hungary: indication of various genotypes. BMC Vet Res.

[46] Astobiza I, Tilburg JJ, Piñero A, Hurtado A, García-Pérez AL, Nabuurs-Franssen MH (2012). Genotyping of *Coxiella burnetii* from domestic ruminants in northern Spain. BMC Vet Res.

[47] Joulié A, Sidi-Boumedine K, Bailly X, Gasqui P, Barry S, Jaffrelo L (2017). Molecular epidemiology of *Coxiella burnetii* in French livestock reveals the existence of three main genotype clusters and suggests species-specific associations as well as regional stability. Infect Genet Evol.

[48] Bauer B, Prüfer L, Walter M, Ganter I, Frangoulidis D, Runge M (2020). Comparison of *Coxiella burnetii* excretion between sheep and goats naturally infected with one cattle-associated genotype. Pathogens..

[49] Szymańska-Czerwińska M, Galińska EM, Niemczuk K, Zasępa M (2013). Prevalence of Coxiella burnetii infection in foresters and ticks in the South-Eastern Poland and comparison of diagnostic methods. Ann Agric Environ Med.

[50] Bielawska-Drózd A, Cieślik P, Żakowska D, Głowacka P, Wlizło-Skowronek B, Zięba D (2018). Detection of *Coxiella burnetii* and *Francisella tularensis* in tissues of wild-living animals and in ticks of north-West Poland. Pol J Microbiol.

[51] Pandit P, Hoch T, Ezanno P, Beaudeau F, Vergu E (2016). Spread of *Coxiella burnetii* between dairy cattle herds in an enzootic region: modelling contributions of airborne transmission and trade. Vet Res.

[52] Bielawska-Drózd A, Cieślik P, Mirski T, Gaweł J, Michalski A, Niemcewicz M (2014). Prevalence of *Coxiella burnetii* in environmental samples collected from cattle farms in eastern and Central Poland (2011–2012). Vet Microbiol.

[53] Tilburg JJ, Rossen JW, van Hannen EJ, Melchers WJ, Hermans MH, van de Bovenkamp J (2012). Genotypic diversity of *Coxiella burnetii* in the 2007-2010 Q fever outbreak episodes in the Netherlands. J Clin Microbiol.

[54] Klaassen CH, Nabuurs-Franssen MH, Tilburg JJ, Hamans MA, Horrevorts AM (2009). Multigenotype Q fever outbreak, The Netherlands. Emerg Infect Dis.

